# Pandemic Influenza, Reopening Schools, and Returning to Work

**DOI:** 10.3201/eid1403.080026

**Published:** 2008-03

**Authors:** Martin I. Meltzer

**Affiliations:** * Centers for Disease Control and Prevention, Atlanta, Georgia, USA

**Keywords:** Influenza pandemic, mathematical models, reopening schools, returning to work, commentary

## Abstract

In this issue of Emerging Infectious Diseases, Victoria Davey and Robert Glass present a paper (*1*) in which they consider the question of when to “switch off” community-based interventions designed to reduce the spread of pandemic influenza. These authors attempt to answers questions such as when it would be optimal to reopen schools that have been closed as part of a nonpharmaceutical, communitywide influenza mitigation strategy.

## Abstract 2

Pandemic Influenza, Reopening Schools, and Returning to Work

The authors use a mathematical model, previously described in this journal (*2*), to simulate the spread of pandemic influenza throughout a community that represents the US population. This model is similar to another model that was used to examine the effectiveness of closing schools to slow the spread of influenza pandemic (*3*). Both models simulate the spread of influenza by dividing a representative population into households. The models then track each household member with each member having a defined number of random contacts (per day) that are allocated within a network of possible contacts. Once a contact is calculated to have occurred, the probability of influenza transmission is calculated. Also included in the calculations are variables such as influenza incubation and infectiousness periods.

What does the model “say”? Davey and Glass considered what would happen if schools were reopened and community-wide sequestering were halted when influenza cases in a community fall below preset thresholds (e.g., 1, 2, or 3 cases in 7 days). Sequestering strategies would be restarted if the epidemic pandemic resurged and >10 cases occurred in a 7-day period. This “pulsing technique” would reduce the number of days needed to sequester schoolchildren and the community by 6% to 32%. The authors maintain that for a given pandemic scenario, the reduction in days sequestered would not notably affect the number of persons infected. The implication is that reduction in days sequestered will reduce the economic impact and social disruption caused by community-wide, nonpharmaceutical interventions.

Are the results “believable”? As with all mathematical models, some potential technical problems exist. First, almost all models that simulate individual person-to-person influenza transmission use 1 or 2 databases that record the probability of influenza transmission. One database was recorded in the early 1970s in Tecumseh, Michigan (*4*), and the other among ≈400 households across France (*5*). Is it reasonable to use these estimates to simulate influenza transmission in every community, town, city, and metropolis in the United States? Furthermore, the researchers who calculated these transmission probabilities did not actually measure the probabilities of who infected whom. These probabilities were calculated by using a statistical technique known as maximum likelihood estimation. Essentially, this is reverse engineering to find the transmission probabilities that best fit the measured data (number of cases over time). However, as others have demonstrated, it is possible to reverse-engineer several transmission probabilities that fit the data (*6*). Would the results calculated by Davey and Glass appreciably change if another set of transmission probabilities was used?

Other assumptions may also be examined. Typically, as with other pandemic models, the authors model different rates of compliance, but each rate is assumed to remain static for the duration of sequestering (e.g., 50% compliance during 40 days of sequestering). In reality, compliance with sequestering may be more fragmented; e.g., teenagers may stay sequestered during the morning but not so much in the afternoon. Other behavior may change during a pandemic; e.g., people may alter the number and duration of contacts, with degree of alteration changing as the pandemic progresses. These changes in behavior could either reinforce or reduce the effectiveness of nonpharmaceutical interventions (*7**,**8*). Furthermore, compliance can decrease dramatically when an intervention is stopped and then restarted. Crosby provides an excellent example of reduced compliance after an attempt to reintroduce compulsory wearing of facemasks in San Francisco during the 1918 pandemic (*9*).

Are such models useful? Yes, so long as readers accept that the results are illustrative and are not absolutely accurate. The models clearly illustrate the complexities of estimating influenza transmission and the potential success of interventions (i.e., such models require a very large set of variables, many with uncertain values). Perhaps the most useful role of such models is the debate that is stimulated regarding the most appropriate, and most feasible (i.e., most likely to work), set of interventions.

Dr Meltzer is the senior health economist and a Distinguished Consultant at the Centers for Disease Control and Prevention in Atlanta, Georgia, and an associate editor for Emerging Infectious Diseases. His research interests include modeling of potential responses to smallpox as a bioterrorist weapon, examining the economics of vaccinating restaurant foodhandlers against hepatitis A, assessing the economic impact of pandemic influenza, and developing software to help public health officials plan and prepare for the next influenza pandemic.
